# Distinct mitochondrial respiration profiles in pediatric patients with febrile illness versus sepsis

**DOI:** 10.1038/s41390-024-03420-z

**Published:** 2024-08-03

**Authors:** Laura F. Sartori, Elena Tsemberis, Tyne Hernandez, Katherine Luchette, Donglan Zhang, Sumera Farooqi, Jenny Bush, John C. McCann, Fran Balamuth, Scott L. Weiss

**Affiliations:** 1 Department of Emergency Medicine, Children’s Hospital of Philadelphia, Philadelphia, PA USA; 2 University of Pennsylvania Perelman School of Medicine, Philadelphia, PA USA; 3 Department of Anesthesiology and Critical Care Medicine, Children’s Hospital of Philadelphia, Philadelphia, PA USA; 4 Nemours Children’s Health, Wilmington, DE USA; 5 Sidney Kimmel Medical College – Thomas Jefferson University, Philadelphia, PA USA

## Abstract

**Objective:**

Mitochondrial dysfunction, linked to sepsis-related organ failure, is unknown in febrile illness.

**Methods:**

Prospective study of children in an Emergency Department (ED) with febrile illness or without infection (ED controls); secondary analysis of ICU patients with sepsis or without infection (ICU controls). Mitochondrial oxygen consumption measured in peripheral blood mononuclear cells using respirometry, with primary outcome of spare respiratory capacity (SRC). Mitochondrial content measured as citrate synthase (CS: febrile illness and ED controls) and mitochondrial to nuclear DNA ratio (mtDNA:nDNA: all groups).

**Results:**

SRC was lower in febrile illness (6.7 ± 3.0 pmol/sec/10^6^ cells) and sepsis (5.7 ± 4.7) than ED/PICU controls (8.5 ± 3.7; both *p* < 0.05), but not different between febrile illness and sepsis (*p* = 0.26). Low SRC was driven by increased basal respiration in febrile illness and decreased maximal uncoupled respiration in sepsis. Differences were no longer significant after adjustment for patient demographics. Febrile illness demonstrated lower CS activity than ED controls (*p* = 0.07) and lower mtDNA:nDNA than both ED/PICU controls and sepsis (both *p* < 0.05).

**Conclusion:**

Mitochondrial SRC was reduced in both febrile illness and sepsis, but due to distinct mitochondrial profiles and impacted by demographics. Further work is needed to determine if mitochondrial profiles could differentiate febrile illness from early sepsis.

**Impact statement:**

- Mitochondrial dysfunction has been linked to organ failure in sepsis, but whether mitochondrial alterations are evident in febrile illness without sepsis is unknown.
- In our study, while mitochondrial spare respiratory capacity (SRC), an index of cellular bioenergetic reserve under stress, was reduced in children with both febrile illness and sepsis compared to children without infections, low SRC was driven by increased basal respiration in febrile illness compared with decreased maximal uncoupled respiration in sepsis.
- Additional research is needed to understand if distinct mitochondrial profiles could be used to differentiate febrile illness from early sepsis in children.

## Background

Fever is a common presenting complaint to pediatric emergency departments (ED).^1^ While most children recover from fever without complication, a subset will develop sepsis, a life-threatening syndrome of organ dysfunction due to dysregulated host response.^2^ Despite efforts to improve early diagnosis and treatment, sepsis accounts for 11 million annual deaths globally, with a peak incidence of sepsis in early childhood.^3^ Understanding pathologic mechanisms differentiating sepsis from routine fever is necessary to prevent the evolution from uncomplicated infections to sepsis-induced organ dysfunction and mortality.^4^

Mitochondria, a critical source of cellular energy, contribute to organ dysfunction in sepsis through altered oxygen metabolism, accelerated oxidative injury, and cell death.^5^ In adults, low mitochondrial respiration measured in peripheral blood mononuclear cells (PBMC) has been correlated with the severity of organ dysfunction in sepsis,^6^ with improved respiration linked to clinical recovery.^7^ Similarly, among critically ill children treated for sepsis, the persistence of low PBMC mitochondrial respiration is associated with delayed recovery from sepsis-associated organ dysfunction, prolonged systemic inflammation, and an immune paralysis phenotype.^8^ The most notable difference in PBMC mitochondrial respiration between children with sepsis and non-infected controls is reduced spare respiratory capacity (SRC), a marker of cellular bioenergetic reserve available to respond to stress.^8^ However, because immune cell activation is normally accompanied by a shift from oxidative phosphorylation to glycolysis, it is not clear if early changes in PBMC respiration in sepsis are evidence of an adaptive or dysregulated immune response.^9^ To date, there are no data about PBMC mitochondrial function in febrile children without sepsis to help differentiate these possibilities.

In this pilot study, we compared PBMC mitochondrial function in children with uncomplicated fever to critically ill children with sepsis and children treated in either the ED or PICU without concern for infection. We hypothesized that adaptive immune response would present as a decrease in PBMC mitochondrial respiration in children with uncomplicated febrile illness compared to controls, but similar levels compared to children with sepsis. Alternatively, differences in PBMC mitochondrial respiration profiles observed in sepsis compared to children with uncomplicated febrile illness could suggest a unique immune response in sepsis. Such distinct mitochondrial profiles could potentially be useful as a novel biomarker to differentiate routine febrile illness from early sepsis and perhaps identify mitochondrial dysfunction as an early pathogenic mechanism in sepsis.

## Methods

### Study design and setting

We performed a prospective, observational study of children presenting to the Children’s Hospital of Philadelphia (CHOP) ED from November 2020 through March 2022. We included a secondary analysis of children with sepsis and non-infected controls who were enrolled in a prior study in the CHOP Pediatric Intensive Care Unit (PICU) between May 2014 to June 2018, if those subjects consented to future data use.^8^ Both studies were approved by the CHOP Institutional Review Board, and written informed consent was obtained for all participants.

### Population

In the ED, we enrolled a convenience sample of children >56 days to <18 years who had an intravenous catheter (IV) placed during clinical care. Children were eligible for the fever group if they had a measured temperature ≥38.0 °C during the ED encounter or within 6 h prior to ED presentation and no clinical concern for sepsis or evidence of organ dysfunction.

Patients admitted to the PICU with sepsis from the prior study were included as our sepsis cohort, with inclusion criteria as previously described.^8^ Children were eligible for that study if they met criteria for septic shock and multi-organ dysfunction syndrome (MODS), as defined by the International Pediatric Sepsis Consensus Conference.^2^ All patients were under evaluation for infection but did not universally have culture-proven etiologies. Subjects were excluded from the final cohort if an alternative non-infectious etiology for shock and MODS (ex. vasculitis) was discovered.

Children were eligible as controls if they were afebrile without infectious concerns. Similar to the ED fever group, ED Controls were included if they had an IV placed in the ED for clinical care. Sepsis controls were included from the prior study if they were admitted to the PICU for non-infectious concerns. Children weighing <7.5 kg, those with known white blood cell count <0.5 × 10^3^/µL, known primary mitochondriopathy, cyanotic heart disease, pregnancy, and multi-system trauma were excluded from all groups.

### Clinical data

Patient characteristics, vital signs, and treatment course were obtained via medical record review. Length of stay (LOS) was calculated as time from initial hospital intake to discharge. Pediatric Logistic Organ Dysfunction (PELOD) score was calculated, with higher scores indicating increased organ dysfunction and risk for mortality.^10^ Children were categorized as previously healthy if they did not have previously defined complex chronic medical conditions.^11^

### Measurements

A single blood sample (7.4 ml for weight ≤12 kg or 9.2 ml for weight >12 kg) was collected in citrate vacutainers and immediately transferred to our research laboratory for study measurements. Enrollment was limited to weekdays due to laboratory staff availability for time-sensitive study measurements. An identical blood collection strategy was used for sepsis and non-infected PICU controls enrolled in the prior study, except that the timing of blood collection was within 48 h of PICU admission for sepsis and after at least 24 consecutive hours without fever for PICU controls.^8^

### Mitochondrial respiration

PBMCs were isolated from citrated whole blood by density gradient centrifugation using a standard Ficoll–Hypaque density gradient, as previously described.^8^ PBMC cell counts and viability were performed using the Countess II Automated Cell counter (ThermoFisher) with trypan blue exclusion. Median (interquartile range [IQR]) viability was 75% (60–83%) for patients with febrile illness, 85% (73–93%) for sepsis, 73% (55–79%) for ED controls, and 97% (90–100%) for PICU controls (*p* < 0.01). After isolation, the PBMC pellet was re-suspended in Hank’s balanced salt solution (pH 7.40) containing 5.5 mM glucose, 1 mM pyruvate, and 10 mM HEPES, centrifuged a final time at 100 × g for 10 min at 20 °C, and then again re-suspended in the same respiration buffer.

**Fig. 1:**
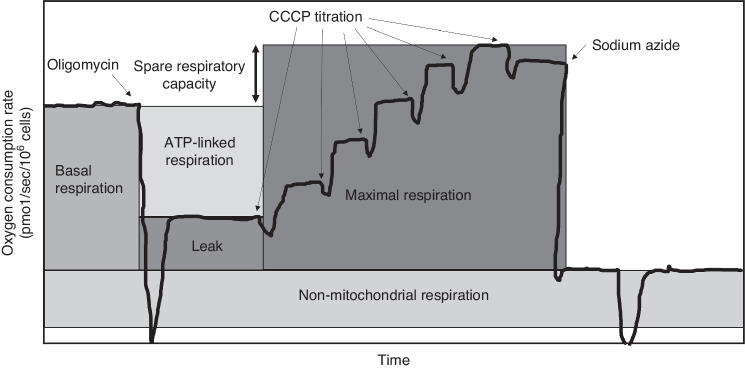
Schematic oxygen tracing of oxygen consumption measured in intact peripheral blood mononuclear cells. The solid black line represents the amount of oxygen present in the chamber over time. Basal oxygen consumption is measured, then oligomycin (ATP-synthase inhibitor) is added to obtain Leak (proton leak after inhibition of ATP synthase). Carbonyl cyanide m-chlorophenylhydrazone (CCCP), an uncoupler, is serially added until no further oxygen consumption is detected to obtain maximal respiration. Non-mitochondrial respiration is obtained by adding sodium azide (electron transport system complex IV inhibitor) and subtracted from other respiration measurements. ATP-linked respiration is calculated as basal minus leak. Spare respiratory capacity (SRC, double-headed arrow) is calculated as maximal respiration minus basal respiration. Adapted from Weiss, et al.^32^.

High-resolution respirometry (HRR) were performed on freshly isolated PBMCs at a concentration of 3–4 × 10^6^ cells/mL using Oroboros respirometry (Innsbruck, Austria). Intact cells were used to measure mitochondrial respiration within a cellular microenvironment that reflects in vivo conditions. Oxygen flux (in pmol O_2_/sec/10^6^ cells), which is directly proportional to oxygen consumption (respiration), was recorded continuously using DatLab software (Oroboros Instruments, Innsbruck, Austria) as previously described.^8,12^ Mitochondrial respiration, including baseline respiration, proton leak after inhibition of adenosine triphosphate (ATP) synthase (leak), and maximal uncoupled respiration through the electron transport system (ETS_max_), were directly measured within intact cells after subtracting residual oxygen consumption following mitochondrial inhibition with antimycin A and sodium azide.^8^ Spare respiratory capacity (SRC) was calculated by subtracting basal respiration from ETS_max_. ATP-linked respiration was calculated by subtracting leak from basal respiration (Fig. 1).

### Mitochondrial content

Mitochondrial content was estimated using citrate synthase (CS) activity measured via spectroscopy, as previously described.^8^ The CS enzyme, part of the tricarboxylic acid cycle, is commonly used as a more practical method to assess mitochondrial content than the gold standard percentage of cell volume.^6^ Measuring mitochondrial content by percentage of cell volume is also prone to intercellular variation.^13^ The CS activity of children enrolled in the ED was normalized per 10^6^ cells and calculated as nmol/min/10^6^ cells. Notably, because CS activity was not normalized per 10^6^ cells as part of the prior PICU study, direct comparison of CS activity was not possible between those enrolled in the ED and PICU. Therefore, mitochondrial DNA relative to nuclear DNA (mtDNA:nDNA ratio) was measured using polymerase chain reaction (PCR) as a second measure of mitochondrial content, as previously described,^8^ because this measure was also available from children in the PICU. Quantitative PCR was performed in triplicate for Thermo Fisher Scientific mtDNA gene ND6 (hs02596879_g1) and nDNA gene Hsp1A1 (hs00359163_s1).

### Outcomes

The primary outcome measure was SRC, previously found to be low in children with sepsis compared to non-infected controls.^8^ Secondary outcome measures were basal, leak, ATP-linked, and ETS_max_ respiration, along with CS activity and mtDNA:nDNA ratio as markers of mitochondrial content. Mitochondrial respiration was normalized to both cell number and, secondarily, to mitochondrial content using CS activity.

### Analysis

Analyzes were performed using STATA (Version 12.1, Stata-Corp, College Station, TX). Data are presented as medians (IQR) or means ( ± SD) for continuous variables and percentages for categorical variables. Continuous data were compared using analysis of variance (ANOVA), Wilcoxon-rank sum, or unpaired t-tests, and percentages were compared using Fisher’s exact test. Correlations were analyzed using Spearman rank. Multivariable linear regression was performed to adjust for age, self-reported race, and previously healthy status due to significant differences in these variables between groups.

## Results

### Study participants

**Fig. 2:**
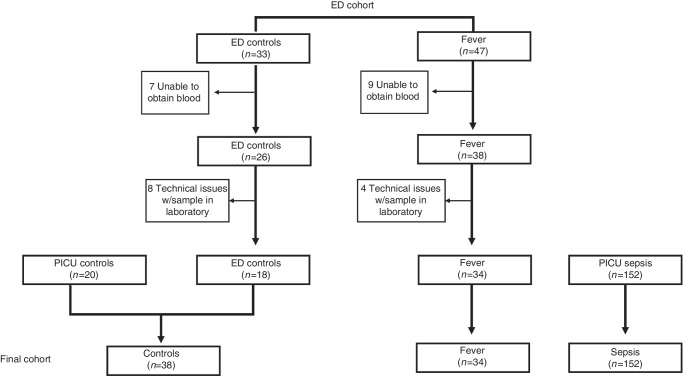
Consort diagram.

Eighty children were enrolled in the ED, with a satisfactory sample available for 52 children: 34 with uncomplicated febrile illness and 18 non-infected controls (Fig. 2). Inability to collect blood after enrollment was the most common reason for exclusion from the final analysis. The 18 ED controls were combined with 20 previously enrolled PICU controls, for a total of 38 non-infected controls. One hundred fifty-two children treated for sepsis in the PICU were also included in this analysis.^8^ The most common reason for IV placement in those with febrile illness was evaluation for Multi-System Inflammatory Syndrome in Children (MIS-C), although none were diagnosed with MIS-C. The most common reason for IV placement in the ED control group was procedural sedation, with samples obtained prior to sedation.

Patient characteristics are presented in Table 1. The median (IQR) age of those with febrile illness was lower than controls or patients with sepsis. Patients with febrile illness were more likely to self-report Black race and and be previously healthy than controls or those with sepsis. Patients with febrile illness experienced the shortest hospital LOS of the three groups, and none in the control or febrile illness groups suffered organ dysfunction during the study encounter or had repeat ED visits with organ dysfunction within 7 days of the study-related encounter.

**Table 1:** Patient characteristics.

	Control *n* = 38	Fever *n* = 34	Sepsis *n* = 152	*P*-value
Age, years (IQR)	10.0 (5.6–14.6)	2.4 (1.3–10)	8.1 (3.4–13.4)	<0.01
Male, *n* (%)	16 (57)	16 (59)	86 (57)	0.97
Race, *n* (%)				<0.01
White	24 (68)	13 (38)	78 (51)	
Black	8 (21)	16 (47)	38 (25)	
Other	3 (11)	0	30 (20)	
Asian	1 (3)	1 (3)	3 (2)	
Unknown	2 (5)	4 (12)	1 (1)	
Ethnicity, *n* (%)				0.43
Hispanic	5 (13)	4 (12)	24 (16)	
Non-Hispanic	32 (84)	30 (88)	121 (80)	
Unknown	1 (3)	0	7 (4)	
Previously healthy, *n* (%)	19 (50)	24 (71)	20 (13)	<0.01
Length of stay, days (IQR)	3.6 (0.3–6.0)	1.2 (0.2–3.3)	18.0 (10.0–34.0)	<0.01
PELOD^a^ Score	0	0	11	<0.01

^a^Pediatric logistic organ dysfunction.

### Mitochondrial respiration normalized to cell number

**Fig. 3:**
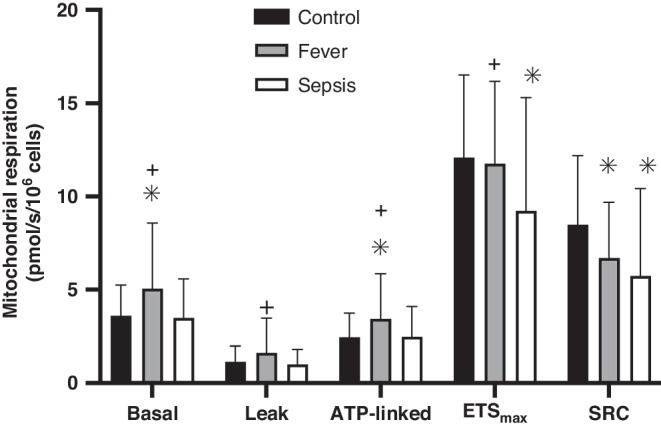
PBMC mitochondrial respiration by group. Bar graph of PBMC mitochondrial respiration. Basal respiration, proton leak after inhibition of ATP synthase (leak), and maximal uncoupled respiration through the electron transport system (ETS_max_), were directly measured using respirometry. ATP-linked respiration was calculated by subtracting leak from basal respiration. Spare respiratory capacity (SRC) was calculated by subtracting basal respiration from ETS_max_. Bars represent mean and error bars represent standard deviation. *p-value ≤ 0.05 compared to controls. +*p*-value ≤ 0.05 compared to sepsis. Fever exhibited higher basal and ATP-linked respiration than controls, but lower SRC. Compared to sepsis, children with fever exhibited higher basal, leak, ATP-linked, and ETS_max_ than sepsis, but no difference in SRC.

The primary endpoint of mean ( ± SD) PBMC mitochondrial SRC did not differ between febrile illness (6.7 ± 3.0Z pmol/sec/10^6^ cells) or sepsis (5.7 ± 4.7 pmol/sec/10^6^ cells; *p* = 0.26). However, SRC was lower than controls (8.5 ± 3.7 pmol/sec/10^6^ cells) for both the febrile illness (*p* = 0.03) and sepsis (*p* < 0.01) groups (Fig. 3, Supplemental Table 1). Secondary respiration endpoints indicated that low SRC was attributable to different respiration profiles in the febrile illness and sepsis groups. In the febrile illness group, low SRC was due to increased basal respiration with preserved ETS_max_ compared to controls. In contrast, low SRC in sepsis was attributable to decreased ETS_max_ with preserved basal respiration. The febrile illness group also exhibited higher leak and ATP-linked respiration compared to controls, which did not differ between the sepsis and control groups (Fig. 3, Supplemental Table 1).

For control patients sampled from the PICU and ED, there were no differences in SRC (9.2 ± 3.8 vs. 7.7 ± 3.6 pmol/sec/10^6^ cells, *p* = 0.22), ATP-linked respiration (2.6 ± 1.2 vs. 2.3 ± 1.4 pmol/sec/10^6^ cells, *p* = 0.35), or ETS_max_ (13.4 ± 4.3 vs. 10.7 ± 4.2 pmol/sec/10^6^ cells, p = 0.06). However, PICU controls did demonstrate slightly higher basal (4.2 ± 1.5 vs. 3.0 ± 1.7 pmol/sec/10^6^ cells, *p* = 0.03) and leak respiration (1.5 ± 0.8 vs. 0.7 ± 0.7 pmol/sec/10^6^ cells., *p* < 0.01) compared to ED controls. Sensitivity analyzes were performed comparing patients with febrile illness and sepsis to each control group individually. Results for separate control groups generally mirrored the larger group, with no change in basal respiration comparisons and a minor change in leak respiration significance for PICU controls versus sepsis (Supplemental Table 2).

After adjusting for differences in age, self-reported race, and comorbidities in multivariable analyzes, the lower SRC observed in febrile illness compared to controls was no longer significant (*p* = 0.09) and remained not different than those with sepsis (*p* = 0.34). The sepsis group continued to have a significantly lower SRC than controls (*p* < 0.01, Supplemental Table 1). ETS_max_ remained not different between febrile illness and controls (*p* = 0.87) and lower in sepsis than controls (*p* < 0.01) but was no longer significantly higher in febrile illness compared to sepsis (*p* = 0.08).

### Mitochondrial content

**Fig. 4:**
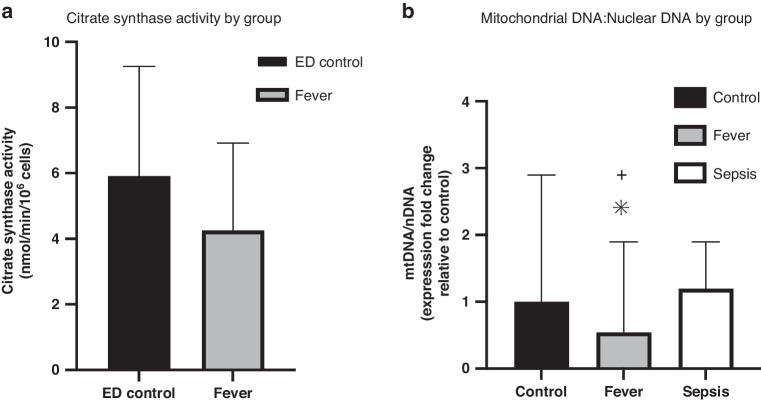
Measures of mitochondrial content by group. **a** Citrate synthase activity by group. Bar graphs of citrate synthase (CS) activity, a measure of mitochondrial content, in control and fever groups. CS was higher in ED controls than fever, though not at a level of significance (p = 0.07). Bars represent mean and error bars represent standard deviation. **b** Mitochondrial DNA:Nuclear DNA by Group. Bar graphs of mitochondrial DNA:nuclear DNA (mtDNA:nDNA), presented as expression fold change in fever and sepsis relative to controls. Expression fold change was lower in fever than in control or sepsis. Bars represent mean and error bars represent standard deviation. *p-value ≤ 0.05 when comparing fever to control groups: +*p*-value ≤ 0.05 when comparing fever to sepsis groups.

CS activity was measured in patients with uncomplicated febrile illness and ED controls. CS activity was lower in those with febrile illness than ED controls, but this difference did not reach statistical significance (4.3 ± 2.7 vs. 5.9 ± 3.3 nmol/min/10^6^ cells, *p* = 0.07; Fig. 4a). Sufficient PBMC residual sample to measure mtDNA:nDNA ratio was available for 23 febrile illness patients, 83 patients with sepsis, and 23 controls. Like CS activity, mtDNA:nDNA ratio was lower in febrile illness than controls (*p* = 0.05) and was also lower compared to those with sepsis (*p* < 0.01; Fig. 4b).

### Mitochondrial respiration normalized to CS activity

**Fig. 5:**
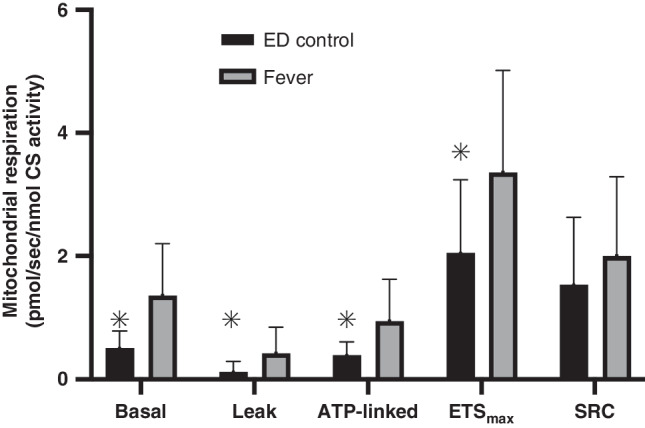
PBMC mitochondrial respiration normalized to mitochondrial content by group. Bar graphs of PBMC mitochondrial respiration (Basal, ATP-linked, leak, ETS_max_, and Spare Respiratory Capacity) adjusted for citrate synthase activity, a marker of mitochondrial content. All measures of mitochondrial respiration except SRC were higher in children with fever compared to controls. Bars represent mean and error bars represent standard deviation. *p-value ≤ 0.05.

Because PBMC mitochondrial content was observed to be lower in febrile illness compared to controls and sepsis, mitochondrial respiration was also analyzed after correcting for CS activity (reported as pmol/sec/nmol CS activity). In this analysis, basal, leak, ATP-linked, and ETS_max_ respiration were higher in patients with febrile illness than in ED controls, while SRC was not different between these two groups (fever, 2.0 ± 1.3 vs. controls, 1.5 ± 1.1 pmol/sec/nmol CS activity, *p* = 0.23; Fig. 5). On adjusted analysis for age, race, and comorbidities, basal, leak, ATP-linked, and ETS_max_ respiration remained higher in patients with febrile illness than in ED controls. SRC remained not different between the two groups (*p* = 0.47).

## Discussion

In this pilot study, we found that SRC, which best differentiated pediatric patients with sepsis from non-infected controls in a previous study,^8^ was also low in ED patients presenting with uncomplicated febrile illness compared to controls. However, the overall PBMC mitochondrial respiration profile included higher basal, leak, ATP-linked, and ETS_max_ respiration in the febrile illness than the sepsis group. These findings suggest distinct profiles of PBMC mitochondrial respiration in uncomplicated febrile illness compared to sepsis rather than a continuum of immune cell activation response. Importantly, given that the decrease in SRC between patients with febrile illness and controls was no longer significant after adjusting for age, race, and comorbidities, at least some of these observed differences in PBMC mitochondrial profile between uncomplicated febrile illness and sepsis may be due to chance differences in demographics between groups. In addition, given that both CS activity and mtDNA:nDNA ratio were lowest in the febrile illness group, it is also possible that part of the mitochondrial profile differences were due to changes in mitochondrial content rather than function.

Prior studies have shown that immune cell activation is accompanied by an adaptive metabolic shift from aerobic oxidative metabolism within mitochondria to accelerated anaerobic glycolysis in the cytosol.^14,15^ Therefore, it is not surprising that mitochondrial SRC in PBMCs is decreased in children both with uncomplicated febrile illness and sepsis compared to non-infection controls. Indeed, we previously demonstrated that the initial fall in mitochondrial SRC on day 1–2 of PICU admission for sepsis was not associated with illness severity or patient outcomes, supporting low SRC as an adaptive immunometabolic response to infection.^8^ Likewise, a reduction in mitochondrial SRC within PBMCs from children with uncomplicated febrile illness could support a similar adaptive immunometabolic response to infection. However, differences in the underlying components of SRC, which is a calculated value, between children with uncomplicated febrile illness and sepsis, suggests an alternative response. ETS_max_, (a calculated value) decreased in sepsis, but not in uncomplicated febrile illness. ETS_max_ is a measure of maximal respiratory capacity when oxygen consumption is uncoupled from ATP synthesis and characterizes the health of the mitochondrial ETS. Therefore, the distinct mitochondrial respiration profiles observed in this study between pediatric patients with uncomplicated febrile illness and sepsis suggest different immunometabolic responses. These findings require further study as a possible novel biomarker to differentiate a self-limited febrile illness from early sepsis and to determine if low ETS_max_ is an early indicator of PBMC mitochondrial dysfunction involved in the pathogenesis of the “dysregulated host response” to sepsis.^2^ Such profiles may eventually aid clinicians in differentiation of uncomplicated febrile illness from early sepsis. For example, a child with low SRC but high ETS_max_, who is otherwise well-appearing, might be identified as a child stable for discharge. In contrast, a child with low SRC and low ETS_max_ may warrant additional monitoring for risk of progression to sepsis.

In analyzes adjusted for age, self-reported race and comorbidities, SRC in fever was not different from controls. One explanation for this change in adjusted analyzes is confounding by patient demographics, as previous literature supports the role of comorbidities as a complicating factor in the evaluation of mitochondrial respiration in children. For example, allergic rhinitis, heart failure, insulin resistance, and trisomy 21 have all been associated with decreased mitochondrial respiration in PBMCs.^16–19^ In contrast, mitochondrial respiration was increased in asthma and autism spectrum disorder, two common comorbidities in children.^20–22^ Age may also play a role, with reported differences in mitochondrial respiration in older (60-80 years) compared to younger adults (18-35 years).^23^ However, change in mitochondrial respiration in healthy children by age has not been defined. Our adjusted analyzes did maintain some similar findings and trends to the non-adjusted analyzes, with no difference in SRC between febrile illness and sepsis and SRC trending lower in febrile illness compared to controls. ETS_max_ similarly trended higher in fever than sepsis in adjusted analyzes, while maintaining lower values in fever compared to controls. These similarities to the unadjusted analyzes suggest our study may have lacked sufficient power to overcome chance differences in demographics, which could be addressed in future studies with a larger or better matched to demographics sample.

When normalized to mitochondrial content, we found that SRC was no longer different between fever and ED controls. All other measures of mitochondrial respiration were higher in fever than controls after normalization to CS activity. We speculate that one mechanism underlying reduced SRC in fever compared to controls may be alterations in mitochondrial dynamics that affect mitochondrial content. For example, studies in adults have demonstrated decreased mtDNA in PBMCs and whole blood from critically ill patients compared to controls. One proposed mechanism of this decrease in mtDNA is excessive oxidative stress during sepsis, with mtDNA particularly susceptible to reactive oxygen species and lacking the enzymes needed for DNA repair.^24^ Two of these studies also demonstrated increased mtDNA with recovery of illness, suggesting a robust mechanism of mitochondrial biogenesis.^25,26^ It is possible that routine fever similarly results in oxidative stress and impaired DNA repair, though the lack of organ dysfunction in our febrile illness group would suggest this stress is not severe or systemic across organ systems. An alternative explanation for decreased mtDNA in the absence of organ dysfunction present in febrile illness is the effect of virus on mtDNA, as previous work has demonstrated mtDNA degradation and release in viral processes.^27–29^ Though viral testing was not uniformly performed in our cohort, it is likely that many children with febrile illness had viral illness.

There are several limitations to this study. First, the single center setting and small sample limit statistical power to assess the impact of potential confounding due to chance differences in patient demographics between groups. Second, because no children progressed to develop sepsis or organ dysfunction, we are not able to comment on the use of mitochondrial respiration as a predictive biomarker. Third, mitochondrial function measurements were limited to respirometry in PBMCs and not tissue-specific measurements. However, PBMCs have been shown as a practical cell type to trend alterations, with good correlation to tissue-specific measurements.^30^ Fourth, PBMCs are a heterogeneous mix of cell types, raising the possibility that respiration reflects variation in immune cell composition across patients, rather than inherent differences in mitochondrial bioenergetics. However, recent evidence suggests influence of immune cell subtypes is not sufficient to account for all measured differences between children with versus without sepsis.^31^ Fifth, differences in methods of blood collection may have contributed to slightly lower cell viability in the febrile illness group, as these samples were usually drawn through a peripheral rather than a central venous or arterial catheter, as in the PICU. Alteration in viability may have resulted in different respiration measurements and explain some differences between ED and PICU controls. Sixth, study groups likely differed in illness timing. Children with fever were likely enrolled earlier in the immune response than those with sepsis, who were enrolled in the PICU on day 1-2 of hospitalization rather than in the ED. Seventh, large variation in measurement within individual groups likely contributed to difficulty discriminating between groups. Finally, data were included from a single timepoint based on clinical presentation and do not account for differences in duration of illness, timing of illness presentation, changes in mitochondrial respiration over time, or baseline variation across individuals.

## Conclusion

Our findings suggest pediatric patients with uncomplicated febrile illness and sepsis both have alterations in PBMC mitochondrial respiration compared to uninfected controls, but in unique ways. Though the ability to draw conclusions from these results is limited by a single timepoint measurement, these distinct PBMC mitochondrial profiles suggest variability in the immunometabolic response in uncomplicated compared to severe infections that result in sepsis. Further characterization of such differences, including studies that better match patient characteristics, is warranted to gain further insight into the mitochondrial pathogenesis of sepsis and/or identify a new high-risk biomarker to differentiate uncomplicated febrile illness from early sepsis.

## Supplementary information

Supplementary Information

## Data Availability

The datasets generated during and/or analysed during the current study are available from the corresponding author on reasonable request.
