# Supplementary material for: Distinct mitochondrial respiration profiles in pediatric patients with febrile illness versus sepsis
Source: Pediatr Res. 2024 Aug 3;97(3):1127–33. doi: 10.1038/s41390-024-03420-z (PMC12055561; doi:10.1038/s41390-024-03420-z)
Supplement: Supplementary file 1 — Supplementary Information [file 41390_2024_3420_MOESM1_ESM.pdf]

Supplemental Table 1

Mitochondrial respiration by group: unadjusted and adjusted analyses

Respiration*	Control n=38	Fever n=34	Sepsis n=152	Control Versus Fever p-value	Control Versus Sepsis p-value	Fever Versus Sepsis p-value	Control Versus Fever Adjusted† p-value	Control Versus Sepsis Adjusted† p-value	Fever Versus Sepsis Adjusted† p-value
**Basal**	3.6 ±1.7	5.1 ±3.5	3.5 ±2.1	0.02	0.78	< 0.01	< 0.01	0.99	0.02
**Leak**	1.1 ±0.8	1.6 ±1.9	1.0 ±0.8	0.15	0.38	< 0.01	0.02	0.31	< 0.01
**ATP-Linked**	2.5 ±1.3	3.4 ±2.4	2.5 ±1.6	0.03	0.93	< 0.01	0.02	0.52	0.17
**ETSmax**	12.1 ±4.4	11.8 ± 4.4	9.2 ± 6.1	0.75	< 0.01	0.02	0.87	< 0.01	0.08
**SRC**	8.5 ±3.7	6.7 ±3.0	5.7 ±4.7	0.03	< 0.01	0.26	0.09	< 0.01	0.34

*Measured in pmol/sec/10^6 cells: data are reported as mean, ± standard deviation
†Comparisons adjusted for age, race, and previously healthy status
Basal respiration, proton leak after inhibition of ATP synthase (leak), and maximal uncoupled respiration through the electron transport system (ETS_{max}), were directly measured using respirometry ATP-linked respiration was calculated by subtracting LEAK from basal respiration. Spare respiratory capacity (SRC) was calculated by subtracting basal respiration from ETS_{max}.

Supplemental Table 2

Mitochondrial respiration by group: comparison of PICU and ED Controls

Respiration*	PICU Control n=20	ED Control n=18	Fever n=34	Sepsis n=152	PICU Control Versus ED Control p-value	PICU Control Versus Fever p-value	ED Control Versus Fever p-value	PICU Control Versus Sepsis p-value	ED Control Versus Sepsis p-value
**Basal**	4.2 ±1.5	3.0 ±1.7	5.1 ±3.5	3.5 ±2.1	0.03	0.28	0.02	0.17	0.31
**Leak**	1.5 ±0.8	0.7 ±0.7	1.6 ±1.9	1.0 ±0.8	<0.01	0.79	0.05	< 0.01	0.16
**ATP-Linked**	2.6 ±1.2	2.3 ±1.4	3.4 ±2.4	2.5 ±1.6	0.35	0.18	0.06	0.66	0.55
**ETSmax**	13.4 ±4.3	10.7 ±4.2	11.8 ± 4.4	9.2 ± 6.1	0.06	0.20	0.40	< 0.01	0.33
**SRC**	9.2 ±3.8	7.7 ±3.6	6.7 ±3.0	5.7 ±4.7	0.22	< 0.01	0.28	< 0.01	0.09

*Measured in pmol/sec/10^6 cells: data are reported as mean, ± standard deviation
Basal respiration, proton leak after inhibition of ATP synthase (leak), and maximal uncoupled respiration through the electron transport system (ETS_{max}), were directly measured using respirometry ATP-linked respiration was calculated by subtracting LEAK from basal respiration. Spare respiratory capacity (SRC) was calculated by subtracting basal respiration from ETS_{max}.

Supplemental Figure 1

Spare Respiratory Capacity (SRC) by Age 1a) All Subjects 1b) By Group

1a)

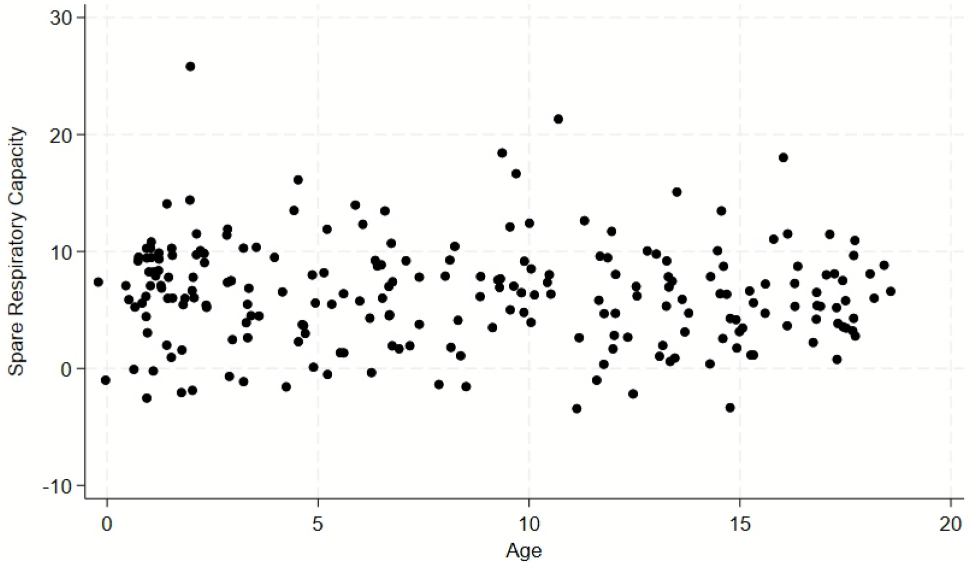

Scatterplot of Spare Respiratory Capacity (SRC) by Age in years, for all subjects. Each dot represents an individual subject. Pearson's correlation coefficient = -0.063.

1b)

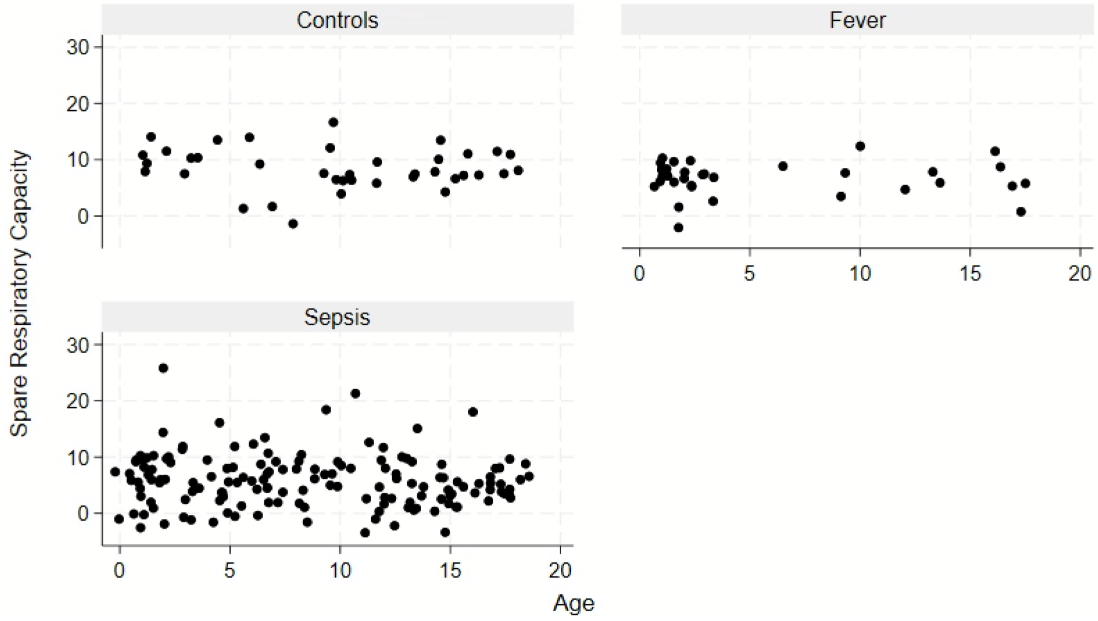

Scatterplot of Spare Respiratory Capacity (SRC) by Age in years, for all subjects. Each dot represents an individual subject. Pearson's correlation coefficient for Controls = -0.11; for Fever= -0.02 and for Sepsis= -0.08

Supplemental Figure 2

Spare Respiratory Capacity (SRC) by Presence of Active Fever at Time of Sample Collection in a) All Subjects b) Fever c) Sepsis

2a) All Subjects 2b) Fever 2c) Sepsis

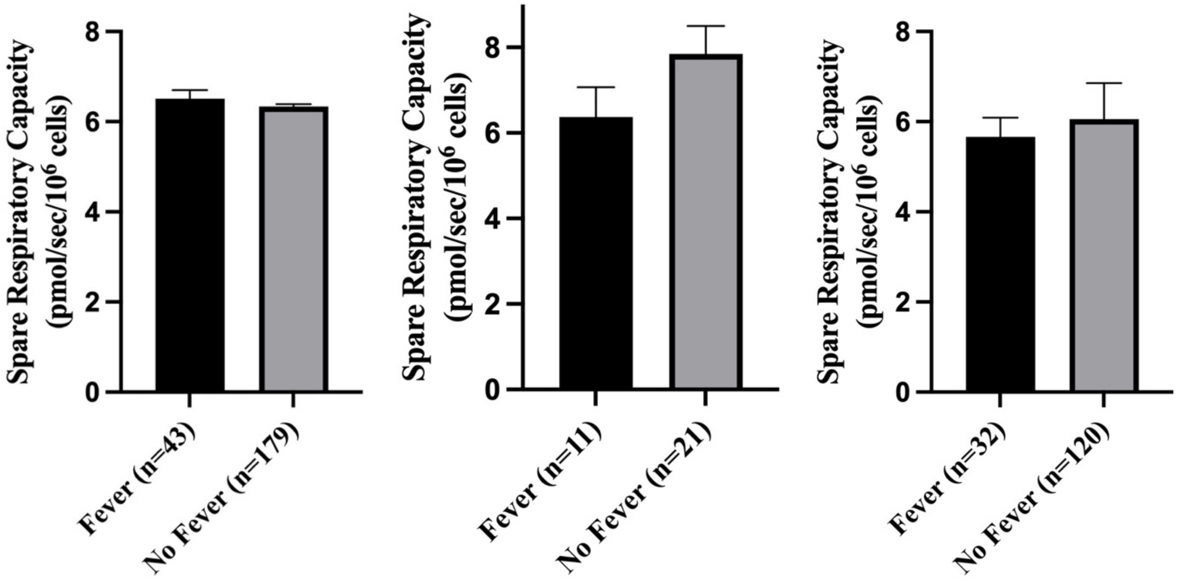

Bar graph of Spare Respiratory Capacity based on presence (Fever) or absence (No Fever) of fever at time of sample collection. Bars represent mean and error bars represent standard deviation. Data was missing from 2 subjects in the fever group. There was no statistical difference between presence or absence of active fever in all subjects (p=0.68), fever (p=0.24), or sepsis (0.46)
